# Mechanical Angle and Its Relationship to Intercondylar Fossa Stenosis: An Anatomical Donor Study

**DOI:** 10.7759/cureus.84882

**Published:** 2025-05-27

**Authors:** Luke Reardon, Collin Archibald, Anne Marie Zeller, Adam Kolatorowicz

**Affiliations:** 1 Orthopedics, Lincoln Memorial University-DeBusk College of Osteopathic Medicine, Knoxville, USA; 2 Osteopathic Medicine, Lincoln Memorial University-DeBusk College of Osteopathic Medicine, Knoxville, USA; 3 Anatomical Sciences, Lincoln Memorial University-DeBusk College of Osteopathic Medicine, Knoxville, USA

**Keywords:** anatomical donor, anterior cruciate ligament (acl), intercondylar fossa shape, intercondylar fossa stenosis, mechanical angle, q-angle

## Abstract

Background

Current literature reveals several anatomic variations that can predispose patients to non-contact anterior cruciate ligament (ACL) injuries. The intercondylar fossa (ICF) plays a crucial role in both acute and chronic knee injuries, housing critical structures like the ACL and posterior cruciate ligament (PCL). This anatomical donor study investigates the relationship between mechanical angle, ICF stenosis, and the Q-angle to understand the potential predisposition to ACL injury.

Methods

A retrospective anatomical donor study was conducted in the anatomical laboratory at Lincoln Memorial University-DeBusk College of Osteopathic Medicine (LMU-DCOM). The study utilized 27 formalin-fixed whole-body cadaveric donors (16 male, 11 female), from which 39 knees were dissected and analyzed. Standardized dissection techniques were employed to expose relevant osseous and soft tissue structures. Measurements of mechanical axis angle, quadriceps (Q) angle, ICF morphology, and femoral and tibial bone dimensions were obtained using consistent anatomical landmarks. All measurements were recorded in a controlled laboratory environment to minimize variability and ensure data reliability.

Results

Logistic regression analysis showed that mechanical angle, Q-angle, and ICF shape were not collectively predictive of ICF stenosis (p > 0.05). However, a significant sex difference in mechanical angle (p = 0.0041) suggests unique biomechanical factors. A negative correlation between Q-angle and mechanical angle (-0.2452) highlighted a complex interplay. Chi-squared analysis indicated trends in ICF shape variation between sexes. Interobserver analysis demonstrated consistent measurements.

Conclusions

Mechanical angle, Q-angle, and ICF shape collectively failed to predict ICF stenosis. However, sex-specific differences in mechanical angle may contribute to ACL injury risk. These findings may indicate the importance of biomechanical variations in injury prevention strategies. Future research should consider age-diverse and longitudinal studies to better understand these dynamics.

## Introduction

Knee injuries, specifically the anterior cruciate ligament (ACL), are prevalent in both the general population and athletes, with an annual incidence of 0.03% in the general population and significantly higher rates of 0.21% to 3.67% among professional athletes [1,2]. One anatomic area of interest in knee injuries is the intercondylar fossa (ICF), or intercondylar notch. The ICF is the area on the posteroinferior aspect of the distal femur between both femoral condyles. Within the fossa are the ACL, posterior cruciate ligament (PCL), anterior and posterior meniscofemoral ligaments, and pericruciate fat. The medial plica, when present, shows a prevalence of 10% clinically, yet 50% on autopsy [3,4]. A study by Davis et al. found that females generally have an ICF, ACL, and PCL compared to males. However, the same study reported that notch width was positively correlated with the size of both the ACL and PCL [5]. Based on these findings, the authors suggested that males may be at increased risk of knee injury due to larger ligament sizes. When combined with stenotic changes, this may result in a reduced notch index, potentially increasing injury risk [5]. Further anatomical differences have been observed in femoral alignment. A study by Deakin et al. reported an average femoral mechanical angle (FMA) of 5.7°, with a standard deviation of 1.2° and a range from 2° to 9° [6]. Males were found to have significantly larger FMA angles than females (p < 0.001), suggesting sex-specific variations that may influence knee biomechanics [6].

ACL injury, particularly rupture, can significantly affect quality of life. A 2023 study estimated the annual incidence of ACL injury in the United States to be approximately one in 3,500 individuals [7]. Another study found that a narrow notch width index (NWI), used as an indicator of stenosis, was significantly associated with increased risk of both unilateral and bilateral ACL ruptures in pediatric populations. Patients with bilateral ACL ruptures had statistically lower NWI measurements compared to controls [8].

Given the high incidence of ACL injury, there is a need to identify anatomical risk factors that may predispose individuals to such injuries. Although females have a higher rate of ACL tears, the underlying causes remain unclear. A study from 2015 found that a narrow ICF and a high alpha angle were significantly associated with ACL tears [9].

Narrowing of the ICF can be secondary to various causes, including congenital narrowing, calcification of the surrounding structures, or changes in the mechanical angle. Hirtler et al. found significant changes occur in the femur and ICF throughout life secondary to degenerative changes and calcifications, such as with arthritis [3]. They found these changes result in more narrowing distally and widening more proximally, meaning that the bicondylar width will increase with age, and the ICF index will decrease or narrow with age. They also noted changes in the shape of the ICF as “A”-shaped (narrow and triangular at the base) structures became omega-shaped (wider, rounded, and resembling the Greek letter Ω) in the later stages of life as calcifications built up. LaPrade et al. categorize distinct intercondylar notch shapes - “A”-shaped (narrow, triangular), “U”-shaped (wider and more uniform), and omega-shaped (rounded, resembling the Greek letter Ω) - noting their significance in understanding knee joint morphology and potential implications for ligamentous injuries [10].

These age-related anatomical changes increase the risk of ACL rupture, although not all of the changes cause damage. More data would be needed to determine whether age, genetics, sex, or stresses such as early specialization in competitive athletics could accelerate or impact these findings. Moreover, these changes over the anterior notch can increase the risk of ACL injury regardless of age or sex [11]. Due to an existing correlation between the stenosis of the ICF and the increased risk of ACL injury in previous literature, finding a correlation between this change and mechanical angle can improve knowledge of predisposing factors. Research has shown that sharper mechanical angles, such as during cutting movements at 90°, 135°, and 180°, significantly increase knee varus moments, which in turn heighten ACL injury risk [12]. Understanding the predisposing factors can then improve early diagnosis of ICF Impingement Syndrome and allow for earlier intervention and improvement in outcomes or reduction in risk of ACL injury.

Acquiring precise limb alignment and joint angular data typically involves utilizing extended radiographs for measurement; however, the project involved conducting physical measurements on each of the donors used in the study.

This study aims to utilize measurements regarding the ICF index along with intercondylar shape, quadriceps angle (Q-angle), and mechanical hip-knee-ankle (HKA) angle to determine if varus or valgus alignment can predispose to stenosis of the ICF, thus making these important variables in ACL injury prevention. Most studies to date have utilized imaging modalities such as MRI or radiographs for measurement of the fossa and associated problems. This study aims to utilize anatomical donors to determine more accurate measurements in situ. Traditional anatomical methods may provide more precise and direct measurements by allowing physical manipulation and direct visualization of tissues, which can sometimes be limited in resolution and context when using imaging modalities like MRI [13]. There are current studies examining the correlation between the size and shape of the ICF and increased stenosis in this region. However, the current studies do not use the mechanical angle to determine its correlation with stenosis of the ICF. This study hypothesizes that mechanical angle, Q-angle, and ICF shape are associated with ICF stenosis in anatomical donors. This study hypothesizes that mechanical angle, Q-angle, and ICF shape are associated with ICF stenosis in anatomical donors.

## Materials and methods

A total of 27 formalin-fixed whole-body donors (16 male, 11 female) were provided by Lincoln Memorial University-DeBusk College of Osteopathic Medicine Anatomical Donation Program and West Virginia University Human Gift Registry. Thirty-nine individual knees were observed (23 male, 16 female). The average age of the donors was 74 years (range: 50-96 years, median: 76, standard deviation: 11.8). All donors had undergone prior dissection unrelated to the structures measured in this study, and all knee capsules were intact at the time of data collection.

The study provided by Cooke et al. set the foundation for assessing lower limb alignment through mechanical angle, correlating it to genu varus and genu valgus [14]. It is determined by a line extending from the center of the femoral head to the medial tibial spine [14]. That line is then compared to the 0° axis formed by the line between the tibial shaft axis running from the midline distal portion of the tibia to the medial tibial spine [14].

The technique was altered to use the center of the patella as the intersection point of the femoral and tibial axes, avoiding the need to reflect the patella and potentially disrupt the angle to expose the tibial spine. The Q-angle is a critical parameter used in orthopedic assessments, specifically in evaluating patellofemoral disorders and knee joint mechanics. It denotes the angle between the quadriceps muscle and the patellar tendon, reflecting the lateral deviation of the force applied by the quadriceps on the patella. In the standing position, the average Q-angle was measured to be approximately 12.3° for the right knee and 12.1° for the left [15]. The methodology includes key measurements: MCWp (width of the medial femoral condyle at the level of the popliteal groove), NWp (intercondylar notch width at the level of the popliteal groove), LCWp (width of the lateral femoral condyle at the level of the popliteal groove), MCWj (width of the medial femoral condyle at the joint line), NWj (intercondylar notch width at the joint line), and LCWj (width of the lateral femoral condyle at the joint line) [10]. These measurements provide insights into specific knee joint dimensions, aiding in the assessment of structural variations and potential associations with knee-related pathologies. The ICF shapes were categorized into “A”-shaped, “U”-shaped, and omega-shaped based on LaPrade et al.'s classification to analyze variations observed in donor specimens [10].

Inclusion and exclusion criteria

Inclusion criteria encompassed donors without prior total hip or knee arthroplasty, including hemiarthroplasties. Donors with structural alterations such as pins or screws affecting measurements were excluded from the dataset. While the presence of mild osteoarthritis or osteophyte growth did not exclude donors, cases with severe osteoarthritic changes, such as significant joint surface lipping or osteophyte formation that interfered with accurate measurements, were excluded. Additionally, donors with dissected knee capsules or disrupted tendons before data collection were excluded due to potential structural alterations impacting accurate angulations. This study employed convenience sampling, utilizing all available donors who met the inclusion criteria during a defined period from March 2023 to December 2023. This ensured a sufficient sample size while adhering to the defined criteria.

Measurements

**Figure 1:**
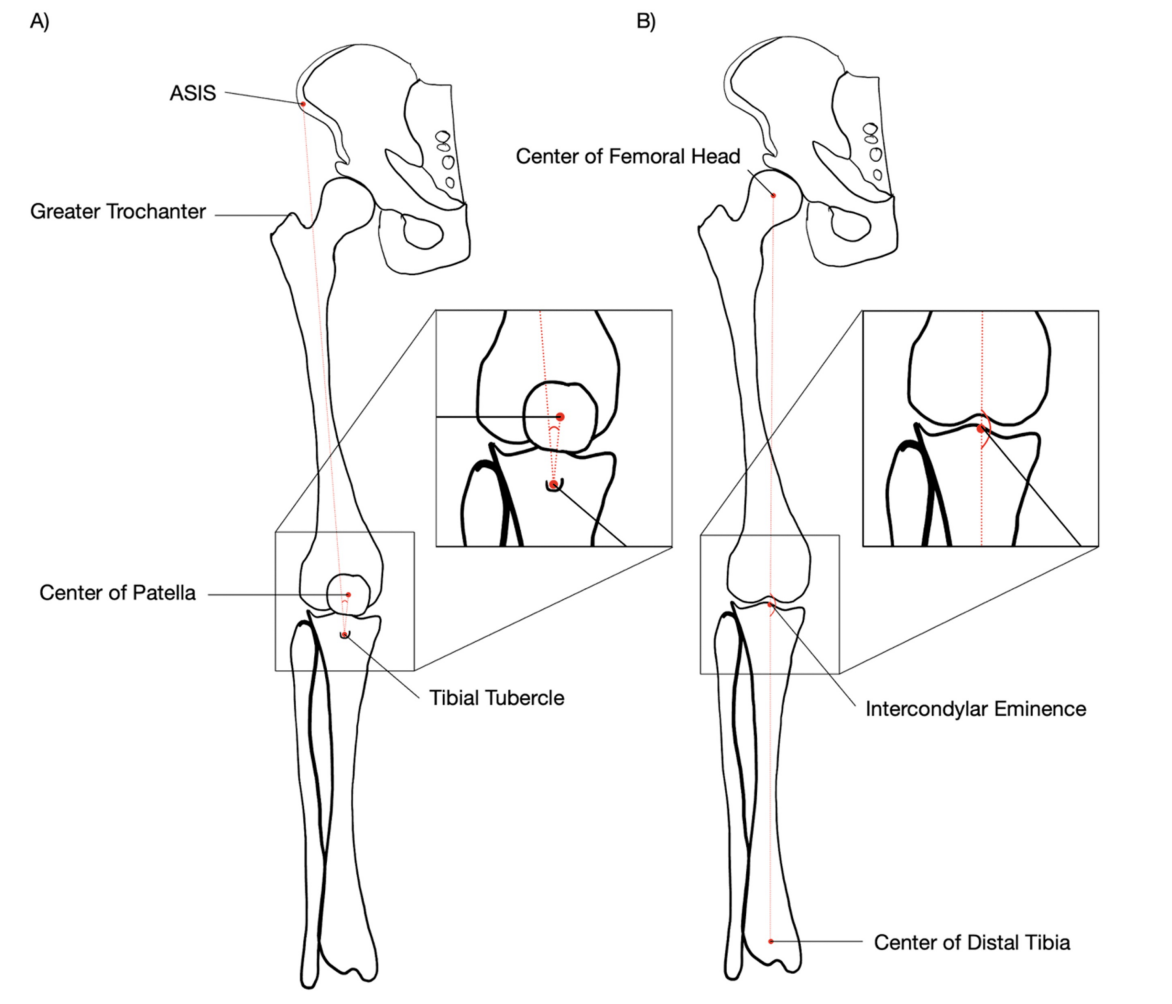
Measurement Mapping for the Quadriceps Angle (A) and Mechanical Angle (B) The left diagram (A) shows the Q-angle, formed between the axis from the anterior superior iliac spine (ASIS) to the patella and from the patella to the tibial tubercle. The right diagram (B) shows the mechanical angle, measured between the femoral axis and tibial axis. Red dotted lines represent the reference axes for these measurements. Image credits: Luke Reardon.

The static Q-angle and mechanical angle were measured before any structural changes were made to ensure unaltered angulation. Both angles were measured while the knee was in full extension in anatomical position. The Q-angle was measured by first isolating the center of the patella, determined by measuring the total height and width with a Mitutoyo Digital Caliper (Model 500-235-30) and dividing it in half. The center of the tibial tubercle was found using the same method as the patella. It was then compared to the center of the patella, setting the 0° axis. The center of the anterior superior iliac spine (ASIS) was then found and compared to the center of the patella; this demonstrated the Q-angle when compared to the 0° axis of the leg (Figure 1A). A goniometer (Baseline, Model 12-1036) was used to measure the angle between the 0° axis and the angle of the quadriceps. The coronal plane angulation of the lower extremities in relation to genu varus and genu valgus (mechanical angles) was used to measure the tibiofemoral angle. This angle is the angle between the shafts of the tibia and femur, and it is measured on the coronal plane. To find the mechanical HKA angle, the femoral head was exposed to identify the center. The femur was placed into an anatomical position while an anterior approach of the hip joint was performed using the digital caliper to identify the height and width of the femoral head. The midline of the distal tibia was found with the digital caliper and marked. A string was placed between the center of the femoral head and the center of the distal tibia. The angulation of the knee was compared with the reference line marked, and the angulation of the knee was measured from the line (Figure 1B).

**Figure 2:**
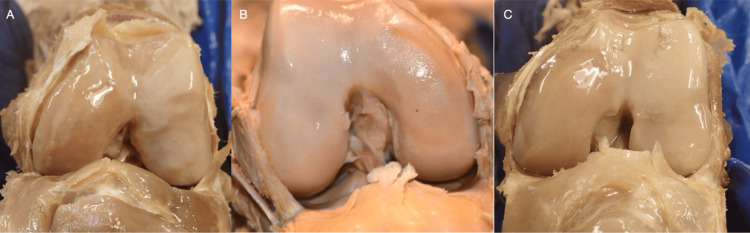
Intercondylar Fossa Shapes This figure showcases intercondylar fossa shapes observed from anterior views of the distal femur with the knee flexed at 90°. The left and right images (A, C) are left knees, while the middle image (B) is a right knee. The left image (A) demonstrates an “A”-shaped intercondylar fossa, the middle image (B) shows an inverse “U”-shaped intercondylar fossa, and the right image (C) shows an omega-shaped intercondylar fossa.

**Figure 3:**
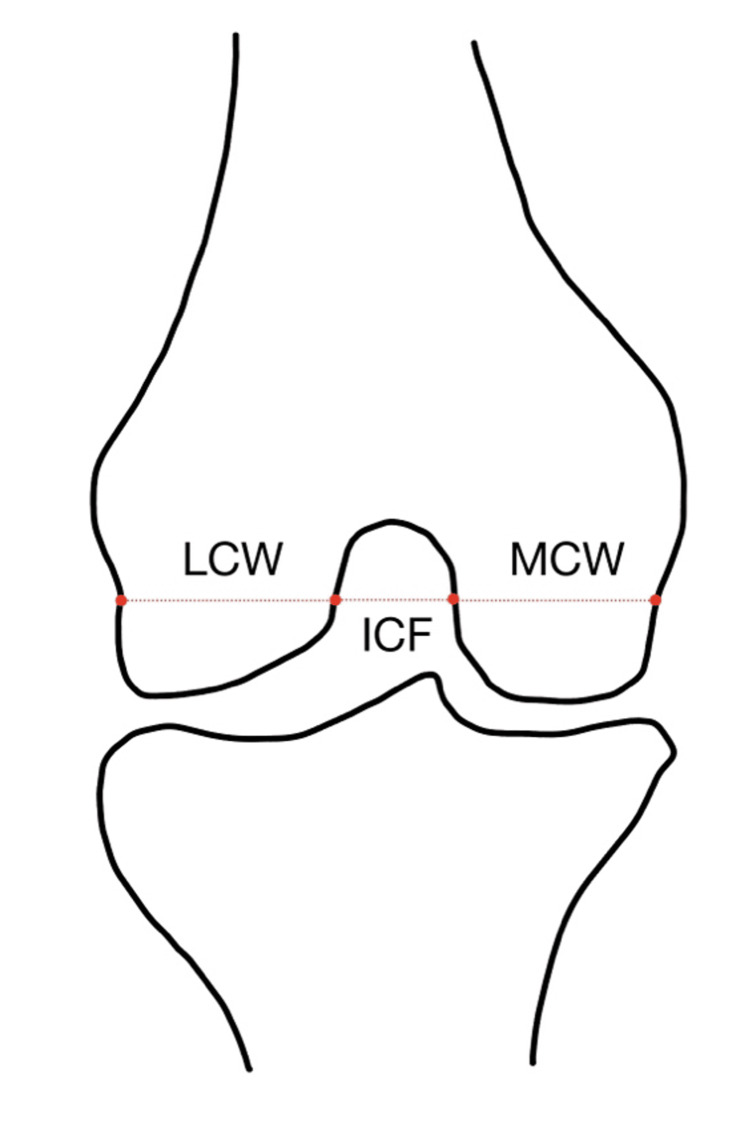
Measurement Mapping for the Lateral Condyle Width (LCW), Intercondylar Fossa Width (ICF), and the Medial Condyle Width (MCW) Diagram of the distal femur indicating the reference line from the popliteal groove on the lateral side and parallel to the joint line (red dotted line) used to measure lateral condyle width (LCW), intercondylar fossa (ICF) width, and medial condyle width (MCW). Image credits: Luke Reardon.

The ICF shape and four bone measurements were made, including lateral condylar width, medial condylar width, ICF width, and bicondylar width. All these measurements were made with a digital caliper in millimeters, recorded to the nearest 0.01mm, with a ±0.04 mm accuracy. The knee joint and bones were exposed by performing a posterior and anterior dissection. First, the knee ICF was identified after performing an anterior approach to remove the quadriceps tendon from the base of the patella, reflecting it inferiorly, and bending the knee to inspect the shape of the ICF. The shape was reviewed, and photos of each ICF were taken. The notch shape was determined based on the current literature, including “A” (Figure 2A), inverse “U” (Figure 2B), and omega-shaped (Figure 2C). To measure the condyles and ICF properly, the structures in the popliteal fossa were dissected for posterior visualization. The popliteus tendon was cut inferiorly, and a horizontal incision was made through the joint capsule on the posterior aspect of the knee. The ICF was then identified and measured with a digital caliper. Landmarks used to define the measurements included the popliteal groove on the lateral side, parallel to the joint line. The bicondylar width was measured at the level of the popliteal groove on the lateral side and parallel to the joint line (Figure 3). The intercondylar notch width was measured in the ICF along the same parallel line as the bicondylar width measurement. Similarly, the medial and lateral condylar widths were also measured on the same parallel line. The ICF notch index was calculated by determining the ratio between the ICF and the bicondylar width of the femur [3].

Data collection

Two independent researchers (LR and CA) conducted measurements simultaneously while blinded to each other’s data. Researchers were trained by a doctoral faculty in anatomy on precise landmarks and locations to measure before commencing the study. Stringent criteria were applied for data validation, and measurements exhibiting discrepancies exceeding two standard deviations were excluded. The data were stored on a password-protected computer on a secure Microsoft Excel (Microsoft® Corp., Redmond, WA) file.

Instruments

To perform the dissections, the following instruments were used: a scalpel, forceps, sharp scissors, gloves, and a string. To measure the mechanical angle and the quadriceps angle, a goniometer was used. To measure the center of the femoral head, distal tibia, and patella, a digital caliper was used.

Statistical analysis

Statistical analyses were performed using RStudio (version 2024.04.1+748, Posit Software, Boston, MA). Both logistic and linear regression analyses were conducted. Logistic regression evaluated the relationship between mechanical angle, Q-angle, ICF shape, and stenosis. Linear regression (Pearson correlation) explored relationships between Q-angle and mechanical angle. The study utilized the maximum number of anatomical donor knees available during the study period, totaling 39 knees (23 male, 16 female). A post-hoc power analysis was conducted to evaluate the statistical power of the sample size. Based on the observed effect size (Cohen's d = 0.49) and the sample distribution, the achieved power was 31%, which is below the conventional threshold of 80% for adequate power. This result indicates that while the sample size represents the total available anatomical donors during the study period, it may limit the ability to detect statistically significant effects. Future studies with larger sample sizes are recommended to confirm these findings.

Model assumption checks

Prior to performing the logistic regression, normality was assessed using Q-Q plots and the Shapiro-Wilk test. All variables showed p-values above 0.05, indicating no significant departure from normality. Variance inflation factors (VIFs) were calculated to assess multicollinearity, with no VIF exceeding 2.0, suggesting no major collinearity issues. Although these steps ensured that basic assumptions for logistic regression were not violated, they did not constitute model validation.

A single logistic regression model was performed with ICF index as the dependent variable and mechanical angle, Q-angle, and ICF shape as predictors. The model did not pass the F test, indicating these three variables cannot be explained by stenosis.

Additionally, independent samples t-tests were conducted for each continuous variable, including Q-angle, mechanical angle, lateral and medial condyle widths, ICF width, and bicondylar width. These t-tests were used to evaluate consistency between the two researchers involved in data collection. For the categorical variable (ICF shape), interobserver agreement was evaluated by comparing classifications made independently by the two observers to ensure consistency in shape categorization. These methods ensured that both continuous and categorical variables were reliably assessed. 

This study was approved by the Institutional Review Board at Lincoln Memorial University-DeBusk College of Osteopathic Medicine under ID FWA00012543 and was classified as Exempt (non-human subjects research). The anatomical donation program provided the formalin-fixed whole-body anatomical donors used in this study. All anatomical donors were managed in accordance with institutional and legal requirements.

## Results

Normality tests were described in the Model Assumption Checks subsection, indicating no major violations of normality or multicollinearity.

Descriptive statistics

Descriptive statistics revealed differences in the Q-angle, mechanical angle, and ICF width between males and females. The average Q-angle for males was 9° and for females 11.5°. The average mechanical angle for males was 7.2° and for females 3.9°. The average ICF width for males in this study was 20.6 mm, while for females, it was 19.5 mm. Studies report average Q-angles of approximately 14° for males and 17° for females, which are higher than those observed in this study [15]. This discrepancy could be attributed to differences in measurement methods, sample populations, or the state of the cadaveric donors used. Formalin fixation, which is known to cause tissue shrinkage and alterations in structural integrity, may have contributed to the reduced Q-angle measurements observed in this study. Deakin et al. reported that males tend to have larger FMA angles compared to females, with the overall mean FMA angle being 5.7° (SD 1.2°) [6]. The intercondylar notch width was reported in previous studies as 19.38 ± 2.90 mm for individuals aged 45-60 years and 18.60 ± 2.36 mm for individuals aged 60 years and older, aligning closely with the findings of this study [3]. Additionally, the advanced age of the donors and potential degenerative changes in the knee joint may have influenced the anatomical measurements, further contributing to the observed differences from living populations. These factors highlight the need for caution when comparing measurements from formalin-fixed donors to those derived from living individuals.

The logistic regression model assessing mechanical angle, Q-angle, and ICF shape as predictors of ICF stenosis was not statistically significant, as it did not pass the F-test. These findings suggest that mechanical angle, Q-angle, and ICF shape are not significantly associated with the presence of stenosis.

Mechanical angle and sex difference

**Figure 4:**
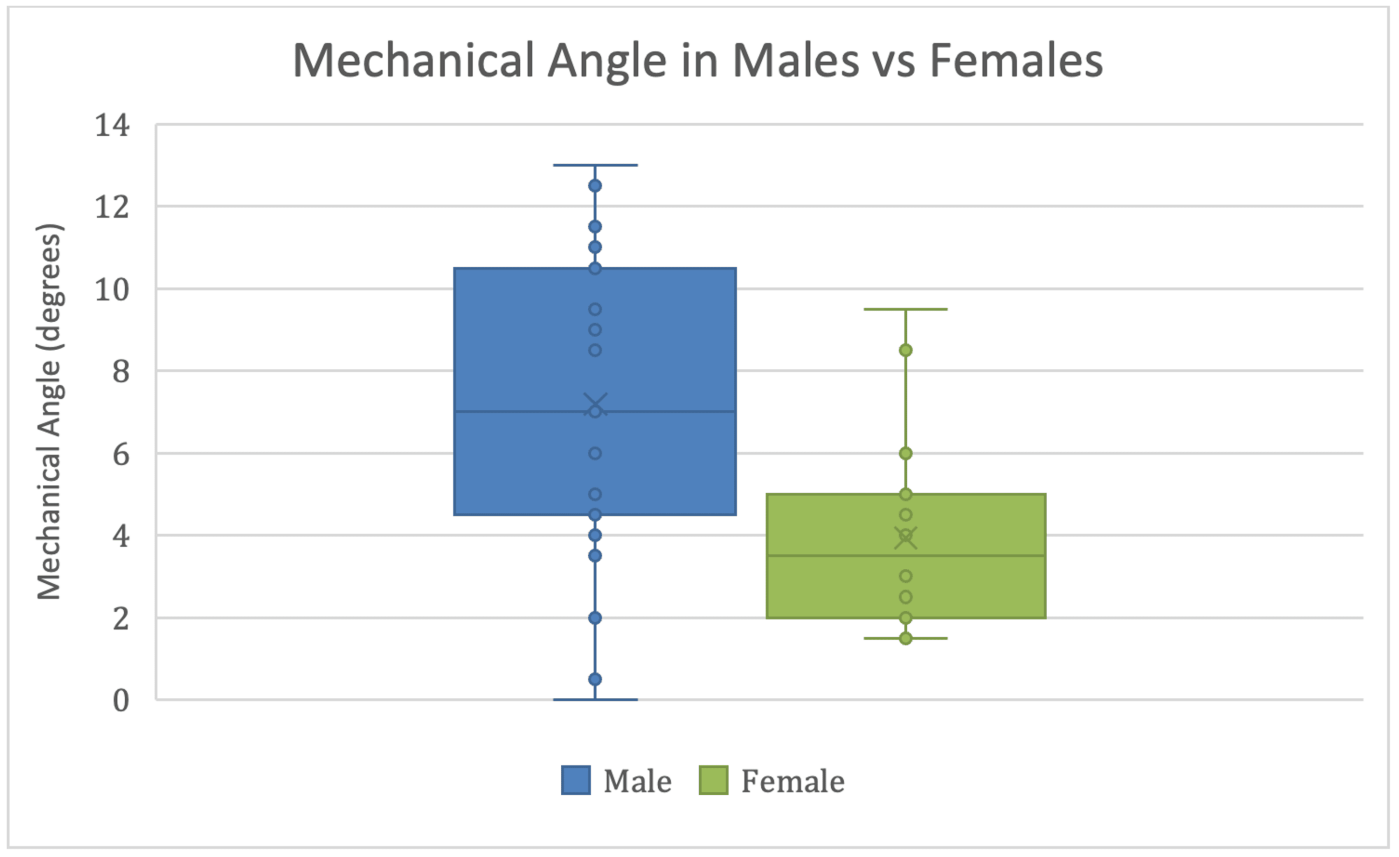
Mechanical Angle in Degrees for Males and Females Box‐and‐whisker plots showing the distribution of mechanical angle measurements for male (blue) and female (green) donors. The median (horizontal line) and mean (x) values indicate that males exhibit a higher and broader range of mechanical angles compared to females, reflecting a significant sex difference (p = 0.0041).

There is a statistically significant difference in mechanical angle between males and females (t37 = 2.03, p = 0.004), with males having an average mechanical angle of 7.20° and females an average mechanical angle of 3.94° (Figure 4).

Q-angle and mechanical angle relationship

**Figure 5:**
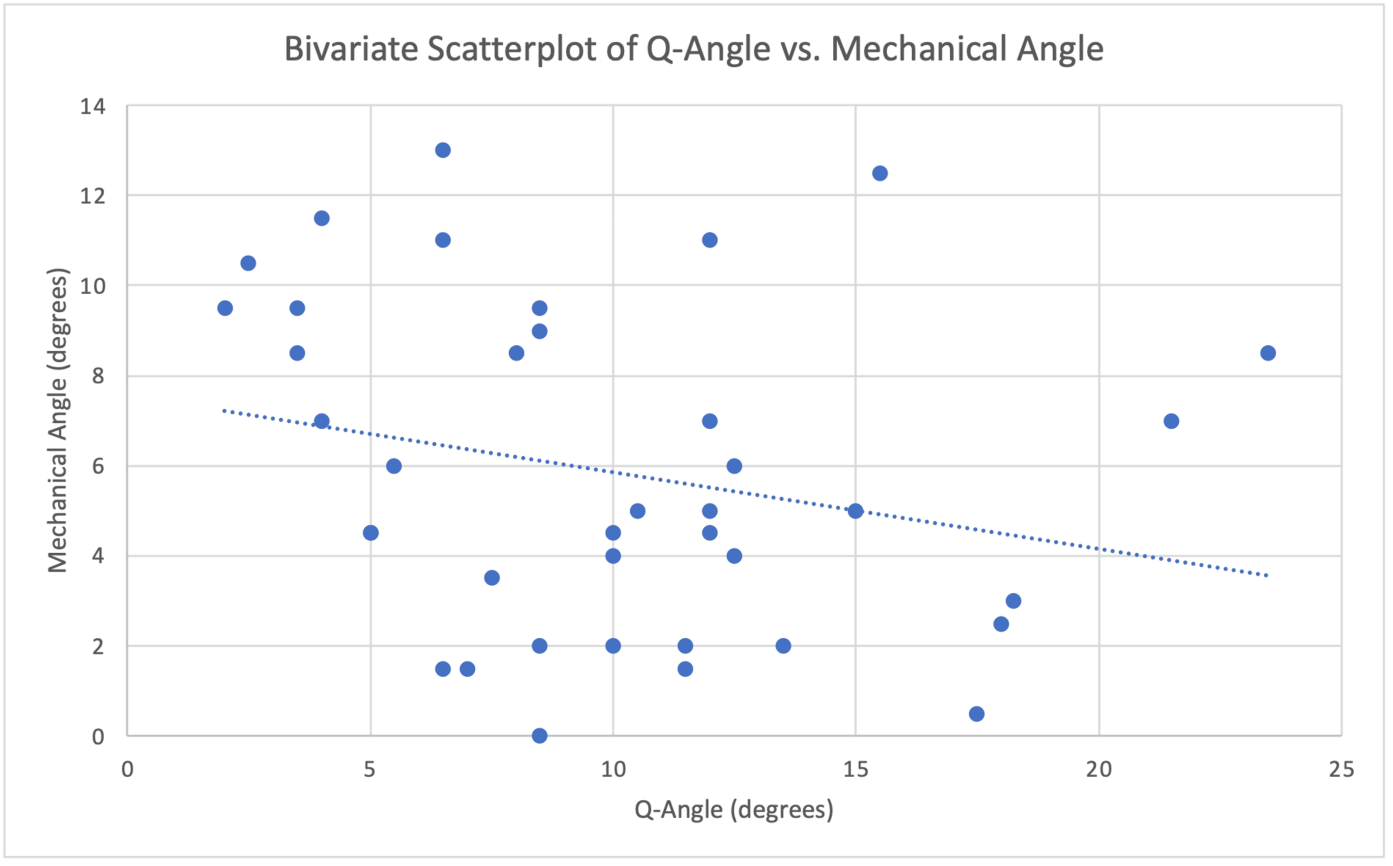
Q-Angle and Mechanical Angle Correlation for Both Males and Females Scatterplot of Q-angle (x-axis) vs. mechanical angle (y-axis) with a fitted regression line (dotted line). A modest negative correlation (r = -0.2452) indicates that as the Q-angle increases, the mechanical angle tends to decrease.

A Pearson correlation analysis demonstrated a modest negative correlation between mechanical angle and Q-angle (r = -0.2452), as illustrated in Figure 5. 

ICF shape and sex difference

**Figure 6:**
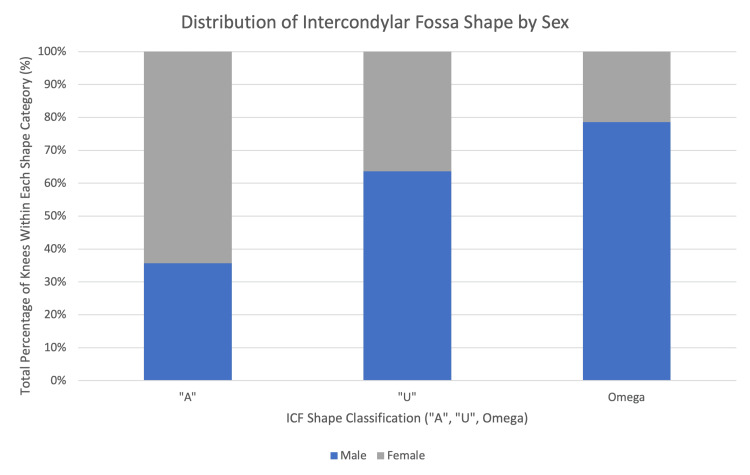
Percentage of Males and Females Categorized by “A,” “U,” and Omega Intercondylar Fossa Shapes Bar graph displaying the percentage of male (blue) and female (gray) donors exhibiting each intercondylar fossa (ICF) shape (“A,” “U,” and omega). Although males show a higher prevalence of “U” and omega shapes, no statistically significant difference between sexes was found (χ² = 5.45, p = 0.06).

The potential association of ICF shape and sex was investigated utilizing a chi-squared analysis. No statistically significant association between sex and ICF shape was found (chi-squared = 5.45, p = 0.06). Table 1 provides a breakdown of the distribution of ICF shapes (“A,” “U,” and omega) between male and female donors, while Figure 6 visually represents these differences. Although not statistically significant, males were observed to have a higher prevalence of “U”-shaped and omega-shaped ICFs, whereas females exhibited a slightly higher proportion of “A”-shaped ICFs. These trends suggest potential anatomical variations that warrant further investigation.

Interobserver reliability analysis

To assess the consistency and agreement between two observers in measuring various parameters related to the study, a series of t-tests were conducted (Table 2).

The obtained p-values from the t-tests indicated no statistically significant differences between the measurements recorded by the two observers for all parameters assessed. These results suggest a high level of agreement and consistency between the observers in quantifying the Q-angle, mechanical angle, lateral and medial condyle widths, ICF width, and bicondylar width.

## Discussion

The comprehensive analysis conducted in this observational study of formalin-fixed whole-body anatomical donors aimed to unravel the intricate relationship between biomechanical factors, specifically Q-angle, mechanical angle, and ICF shape, and the prevalence of stenosis in the ICF. This study found no significant association between global mechanical axis alignment and ICF stenosis, suggesting that overall mechanical angle may not be a primary determinant of notch width in the distal femur. Initially, it was hypothesized that malalignment (varus or valgus deformity) might influence ICF dimensions by altering load distribution across the knee, supported by literature proposing frontal-plane malalignment as a risk factor for ACL injury [7]. The logistic-regression model failed to reach significance, indicating that mechanical angle alone does not meaningfully predict intercondylar-notch stenosis in this older anatomical donor cohort.

A clear sex-specific difference in mechanical angle was observed (mean 7.2° in males vs. 3.9° in females), reflecting the larger FMAs previously reported in men [6]. Although this angular difference did not translate into differential ICF stenosis in this study, it underlines the complex, multifactorial nature of notch narrowing, where bony alignment, notch morphology, and soft-tissue adaptation intersect.

Independent of limb alignment, ICF morphology itself remains an important factor influencing knee stability. Previous research established a strong correlation between narrow intercondylar notch width and increased ACL injury risk. Souryal et al. introduced the NWI, reporting narrower femoral notches in athletes who sustained bilateral ACL ruptures [16]. Shelbourne et al. subsequently confirmed this relationship, showing that smaller notch widths significantly correlated with higher incidences of contralateral ACL tears after ACL reconstruction [17]. These foundational studies support stenosis of the intercondylar notch as a significant anatomical risk factor for ACL injuries.

More recent studies further substantiate the relationship between notch stenosis and ACL pathology. Hoteya et al. observed narrower notches in athletes with bilateral ACL injuries compared to those with unilateral injuries or healthy knees [18]. A meta-analysis by Zeng et al. similarly found significantly lower NWI in ACL-injured individuals than controls, strongly associating narrower notches with increased ACL rupture risk [19]. Notch shape has also been implicated; van Eck et al. noted that knees with ACL tears commonly displayed a stenotic, “A-shaped” morphology compared to broader shapes in intact knees [20]. Collectively, these studies highlight notch geometry, both width and shape, as independent predictors of ACL injury risk, regardless of limb alignment.

Additional evidence from Al-Saeed et al. showed that a stenotic type “A” femoral notch morphology was markedly more common in patients with ACL tears than in those with broader “U” or “omega” notch shapes, emphasizing the importance of notch shape, as opposed to global limb alignment, as an independent anatomical risk factor for ACL injury [21]. When mapped onto the current findings, this supports the premise that shape-based stenosis, rather than coronal malalignment, likely drives true ACL vulnerability.

A non-significant inverse correlation (r = - 0.2452) between Q-angle and mechanical angle was identified, mirroring prior work that links greater valgus knee alignment to smaller FMAs [12]. While small in magnitude, this relationship suggests that dynamic valgus mechanisms, in which Q-angle transiently rises, could affect load across the notch without permanently altering its osseous width; one possible explanation for why alignment did not predict stenosis in this cadaveric model.

The high interobserver reliability reported for all linear and angular measurements strengthens confidence in the quantitative data, indicating that the null findings are unlikely to reflect measurement error. Descriptive statistics showing lower than expected Q-angles and slightly wider mean notch widths compared with living cohorts reinforce the influence of formalin fixation and advanced donor age are factors that may attenuate clinically observable differences.

These results suggest that screening and preventive efforts aimed at ACL injury should prioritize notch morphology, particularly stenotic or “A” type shapes, over assessments of global mechanical alignment. Imaging modalities such as MRI can delineate notch contour and width directly, whereas long-leg standing radiographs that capture the mechanical axis may have limited predictive value for notch stenosis.

This study is subject to several limitations. The achieved post-hoc power of 31% indicates that small true effects may have been missed, especially given the advanced donor age (mean 74 years) and formalin-fixed state of the knees. Anatomical donor measurements taken in non-weight-bearing conditions may diverge from in vivo biomechanics. Post-mortem tissue changes, including altered soft tissue tone, hydration, and morphology due to aging and fixation, may have influenced Q-angle and ICF measurements, limiting generalizability to living populations. Mild osteoarthritic changes could have altered notch dimensions [22]. While every effort was made to ensure measurement accuracy, minor human error during dissection and data collection remains a potential limitation. Additionally, post-mortem tissue changes such as alterations in soft tissue tone, hydration, and morphology resulting from donor age and formalin fixation may have impacted anatomical measurements, potentially limiting the generalizability of these findings to living populations. Finally, the absence of donor clinical histories prevented direct correlation of anatomical findings with clinical ACL injury status. Given the lack of statistical significance in the regression analyses, detailed regression tables were not included, as they would not add meaningful interpretation to the results. Future research incorporating larger, demographic-rich populations and clinical correlations would strengthen these findings.

Despite these limitations, direct anatomical measurements provide high accuracy for assessing bony structures and notch morphology. The current findings contribute valuable insights indicating that limb mechanical alignment alone minimally influences intercondylar notch size, reinforcing the role of intrinsic anatomical factors in determining ACL injury risk.

## Conclusions

These findings reveal a significant sex-specific difference in mechanical angle and highlight a negative modest correlation between Q-angle and mechanical angle. However, the logistic regression model designed to test whether mechanical angle, Q-angle, and ICF shape collectively explained ICF stenosis did not pass the F test. This may indicate that these three factors alone are insufficient to explain stenosis. Further research, particularly with larger and more age-diverse populations, is warranted to investigate additional or alternative factors that may contribute to ICF narrowing and ACL injury risk. Understanding these variations could inform targeted preventive strategies and enhance clinical interventions in knee joint health, ultimately contributing to improved outcomes in orthopedic practice.
